# Correction: Detection and classification of long terminal repeat sequences in plant LTR-retrotransposons and their analysis using explainable machine learning

**DOI:** 10.1186/s13040-024-00417-6

**Published:** 2024-12-30

**Authors:** Jakub Horvath, Pavel Jedlicka, Marie Kratka, Zdenek Kubat, Eduard Kejnovsky, Matej Lexa

**Affiliations:** 1 Faculty of Informatics, Masaryk University, Botanicka 68a, 60200 Brno, Czech Republic; 2 Department of Plant Developmental Genetics, Institute of Biophysics of the Czech Academy of Sciences, Kralovopolska 135, 61200 Brno, Czech Republic; 3 National Centre for Biomolecular Research, Faculty of Science, Masaryk University, Kamenice 5, 62500 Brno, Czech Republic

**Correction: BioData Mining 17**,** 57 (2024)**.

10.1186/s13040-024-00410-z.

Following publication of the original article [1], the authors identified that the funding declaration is not complete. The incorrect and correct statement are given below:

*Incorrect funding statement:*

Financial support for this work was provided by a grant from the Czech Science Foundation number 21–00580 S to EK and ML, 24–11,400 S to EK. The work was supported from the project TowArds Next GENeration Crops, reg. no. CZ.02.01.01/00/22_008/0004581 of the ERDF Programme Johannes Amos Comenius.

*Correct funding statement:*

Financial support for this work was provided by a grant from the the Czech Science Foundation number 21–00580 S to EK and ML, 24–11,400 S to EK, and 22–00364 S to ZK. The work was supported from the project TowArds Next GENeration Crops, reg. no. CZ.02.01.01/00/22_008/0004581 of the ERDF Programme Johannes Amos Comenius.

The original article [1] has been corrected.
