# Supplementary material for: Detection and classification of long terminal repeat sequences in plant LTR-retrotransposons and their analysis using explainable machine learning
Source: BioData Min. 2024 Dec 18;17:57. doi: 10.1186/s13040-024-00410-z (PMC11656987; doi:10.1186/s13040-024-00410-z)
Supplement: Supplementary file 1 — Supplementary Figures Supplementary Figure 1 - LTR sequences comparison - classical approaches. Supplementary Figure 2 - CNN-LSTM model topology Supplementary Figure 3 - DNABERT model topology Supplementary Figure 4 - LTR detection contingency tables Supplementary Figure 5 - LTR detection accuracy Supplementary Figure 6 - Superfamily classification contingency tables Supplementary Figure 7 - Superfamily classification accuracy Supplementary Figure 8 - Family classification contingency tables Supplementary Figure 9 - Family classification accuracy Supplementary Figure 10 - Cross-validation - differences between three models Supplementary Figure 11 - Perturbation analysis Supplementary Figure 12 - gProfiler GOSt analysis of the top 20 GBS model TFBS (LTR task) Supplementary Figure 13 - gProfiler GOSt analysis of the top 20 CNN model TFBS (LTR task) Supplementary Figure 14 - gProfiler GOSt analysis of the top 20 CNN model TFBS (sf task) Supplementary Figure 15 - Main results of explainability analysis (sf classification) Supplementary Figure 16 - DeepExplainer analysis of trained superfamily detection models Supplementary Figure 17 - Results of explainability analysis (family classification) Supplementary Figures Supplementary Table 1 - Tested hyperparameters Supplementary Table 2 - Loss functions Supplementary Table 3 - Cross-validation between models Supplementary Table 4 - Final hyperparameter values Supplementary Files File1_LTR_sequences.fa.gz File2_LTR-negative_sequences.fa.gz File3_non-LTR_counts.tab File4_LTR_species_counts.tab File5_JASPAR_matrices.tab File6_Trained_models.zip File7_Gridsearch_results.json File8_Hyperparameter_sweep.zip File9_Clustering_with_kmers.pdf File10_GBC_SHAP_values.tab File11_CNN_filters.tab File12_kmer_motifs.pdf File13_DNABERT_kmer_SHAP_values.tab File14_CNN_BERT_Grid.pdf [file 13040_2024_410_MOESM1_ESM.pdf]

Supplementary figures

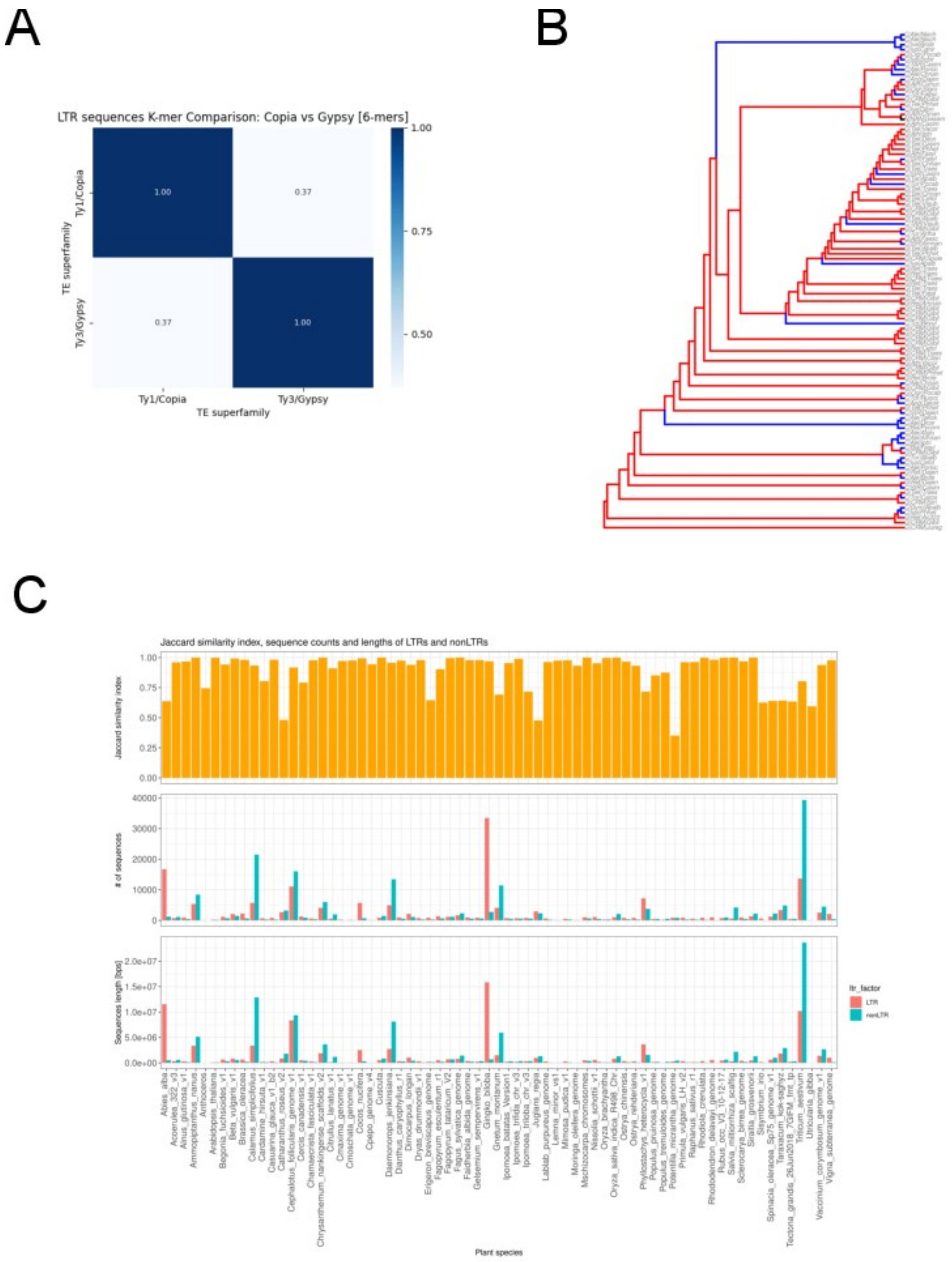

Supplementary Figure 1 - LTR sequences comparison - classical approaches. *Unique k-mers of Ty1/Copia and Ty3/Gypsy LTRs [k = 6; Jaccard Similarity Index (JSI)] (A); Dendrogram constructed from JSI (B); and k-mers occurrence comparison in LTRs and corresponding non-LTR sequences from the genomes of relevant plants as controls (C).*

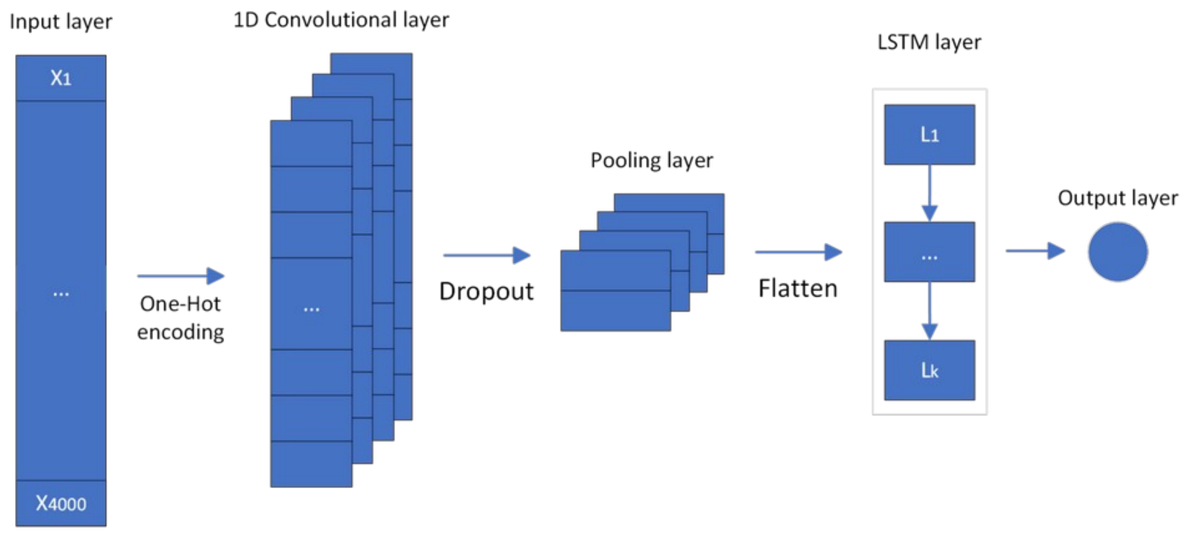

Supplementary Figure 2 - CNN-LSTM model topology.

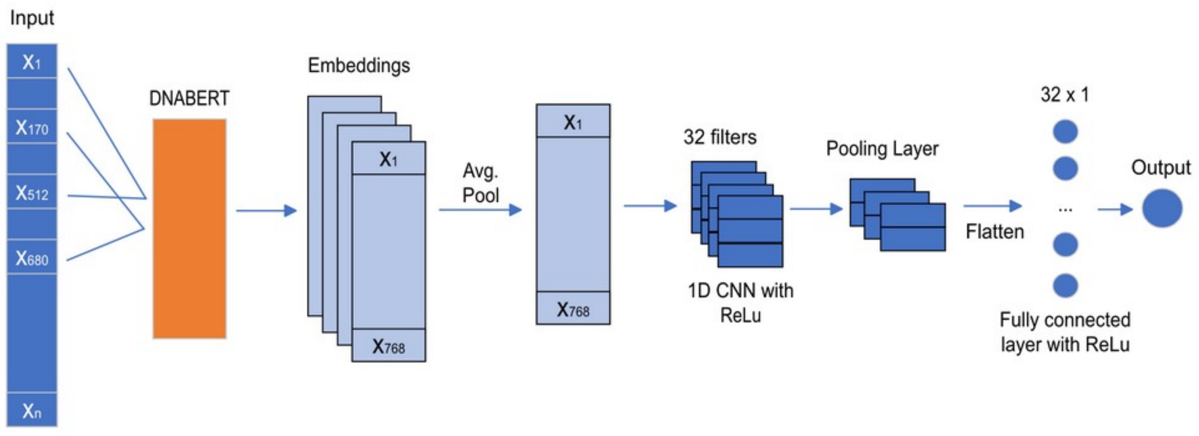

Supplementary Figure 3 - DNABERT model topology.

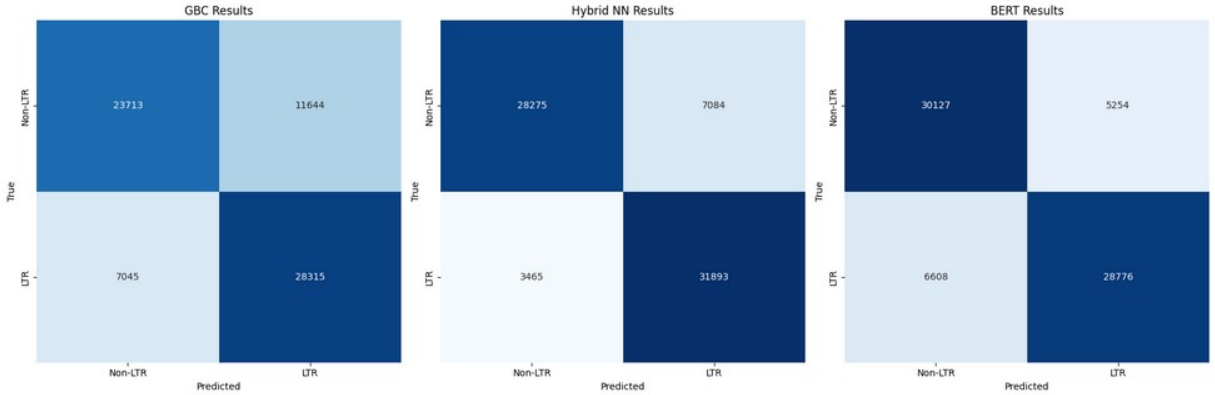

Supplementary Figure 4 - LTR detection contingency tables. *True and false positives/negatives for the three models (GBC, CNN-LSTM and DNABERT) in the LTR binary classification task. The numbers in the contingency table cells are counts of classified LTRs for each combination of attributes.*

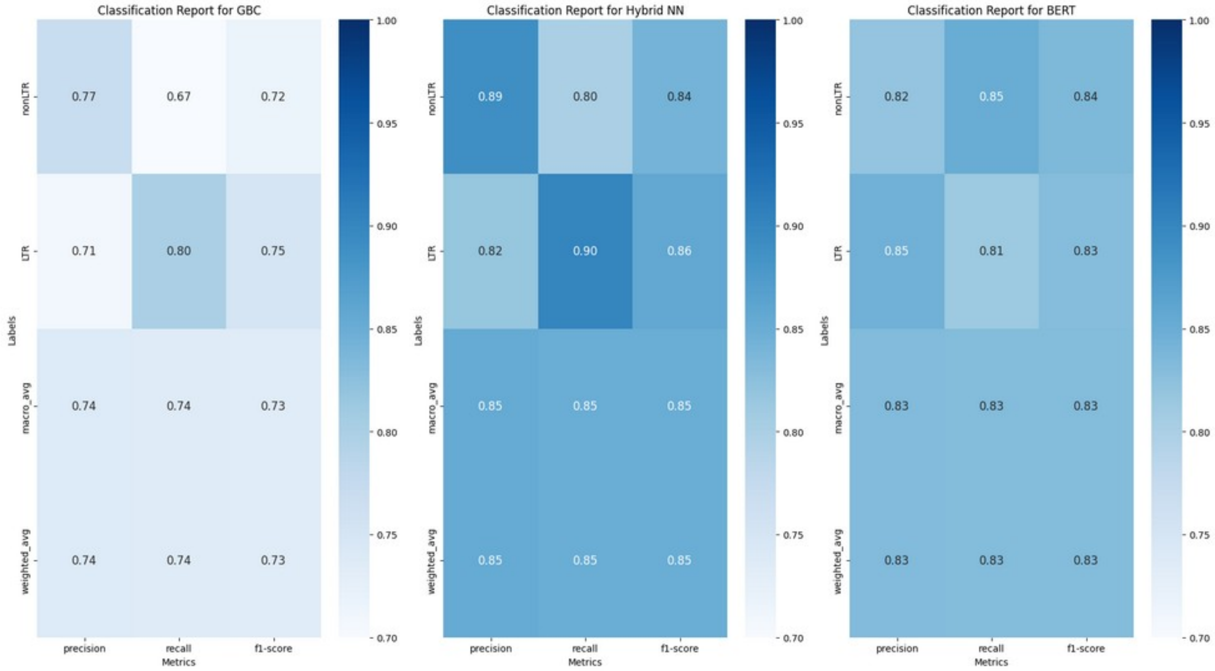

Supplementary Figure 5 - LTR detection accuracy. *Accuracy characteristics (precision, recall and F1 value) calculated from values in Supplementary Figure 1.*

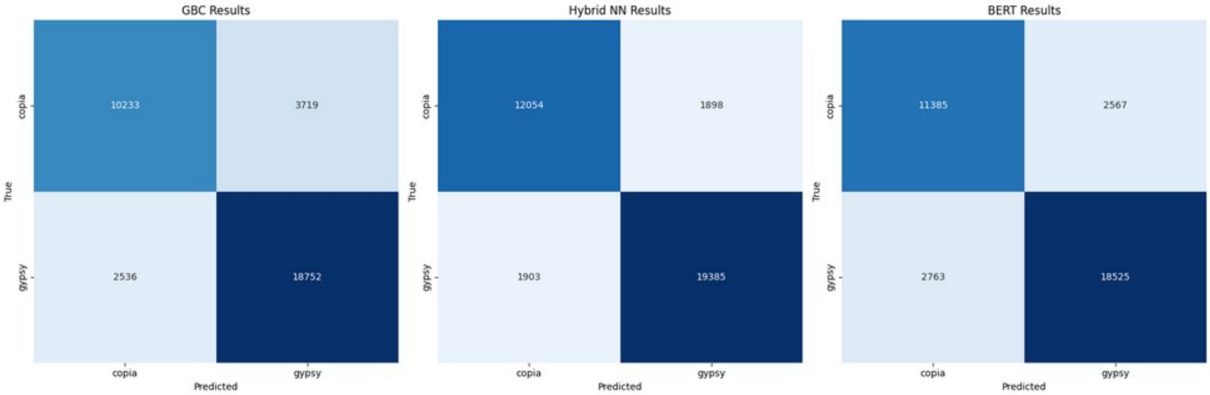

Supplementary Figure 6 - Superfamily classification contingency tables*. True and false positives/negatives for the three models (GBC, CNN-LSTM and DNABERT) in the LTR binary classification task. The numbers in the contingency table cells are counts of classified LTRs for each combination of attributes.*

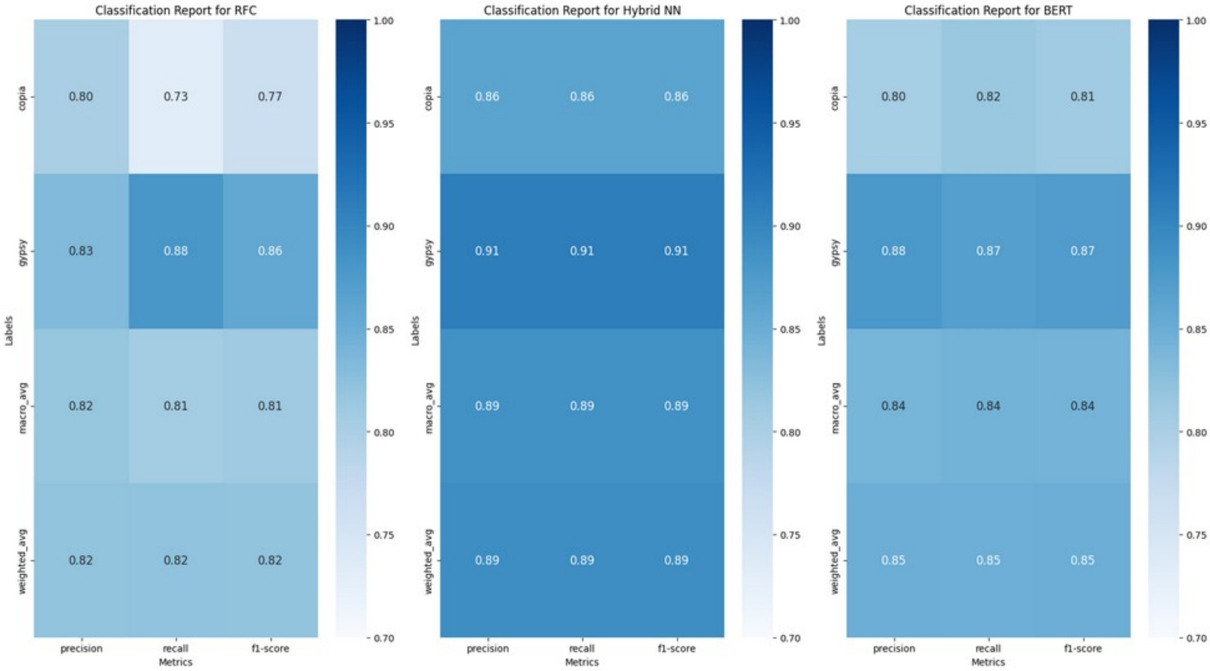

Supplementary Figure 7 - Superfamily classification accuracy*. Accuracy characteristics (precision, recall and F1 value) calculated from values in Supplementary Figure 1.*

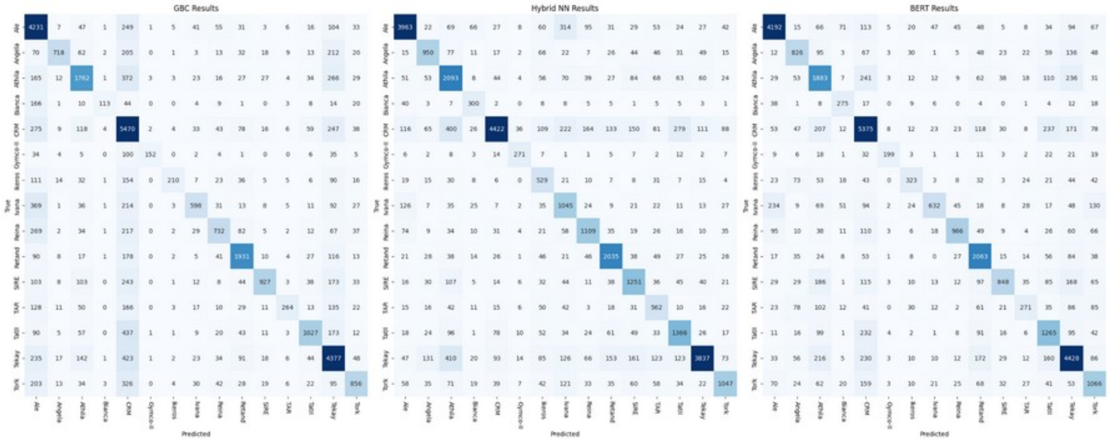

Supplementary Figure 8 - Family classification contingency tables*. True and false positives/negatives for the three models (GBC, CNN-LSTM and DNABERT) in the LTR binary classification task. The numbers in the contingency table cells are counts of classified LTRs for each combination of attributes.*

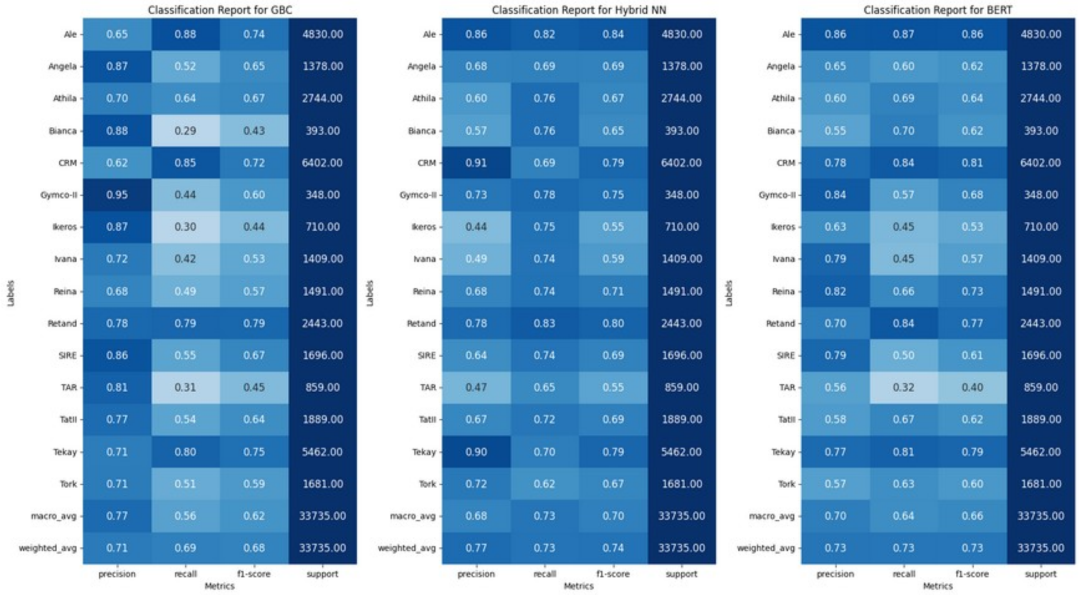

Supplementary Figure 9 - Family classification accuracy*. Accuracy characteristics (precision, recall and F1 value) calculated from values in Supplementary Figure 1.*

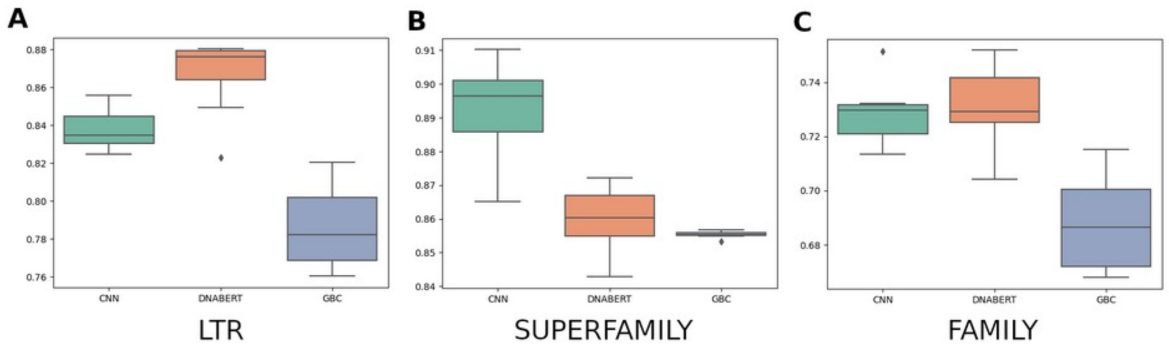

Supplementary Figure 10 – Cross-validation - differences between three models. *Boxplot of the 10-fold cross validation results on the three models in various classification tasks: A) LTR classification; B) superfamily classification; and C) family classification.*

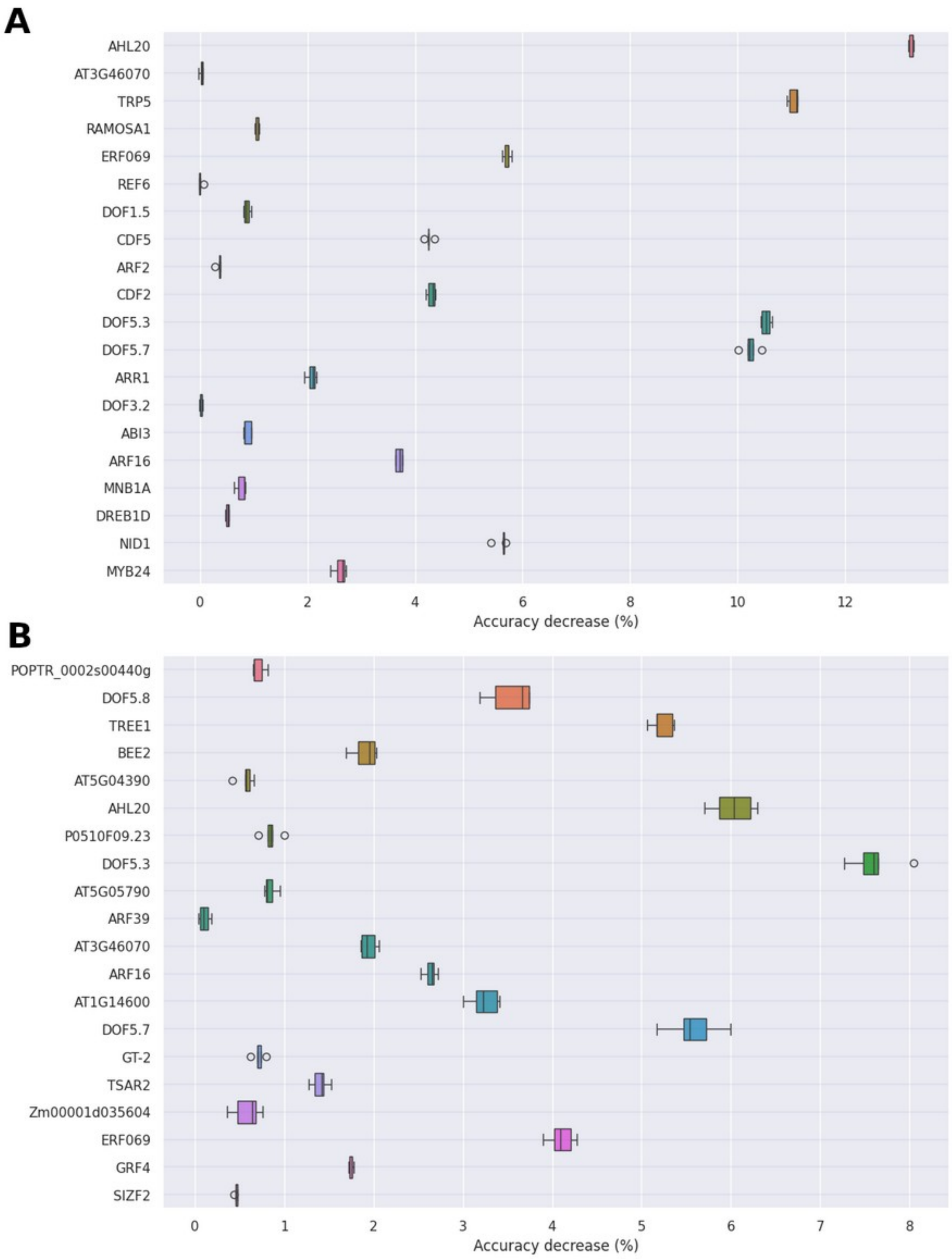

Supplementary Figure 11 – Perturbation analysis. Results of the perturbations done on top 20 TFBS motifs identified as significant by the SHAP algorithm. Plots show decrease of accuracy of the model when data for one TFBS is randomized in the input.

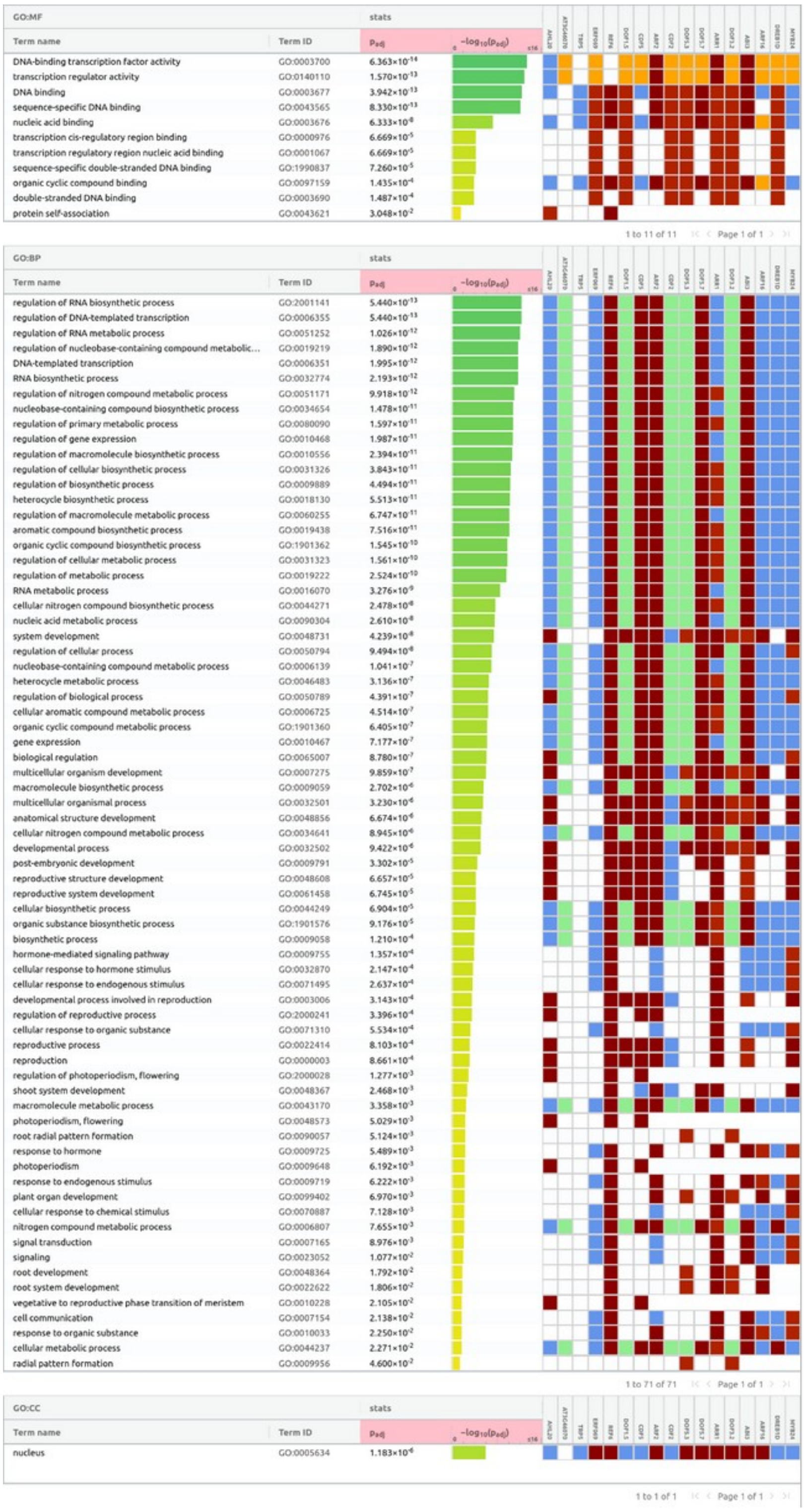

Supplementary Figure 12 - gProfiler GOSt analysis of the top 20 GBS model TFBS (LTR task).

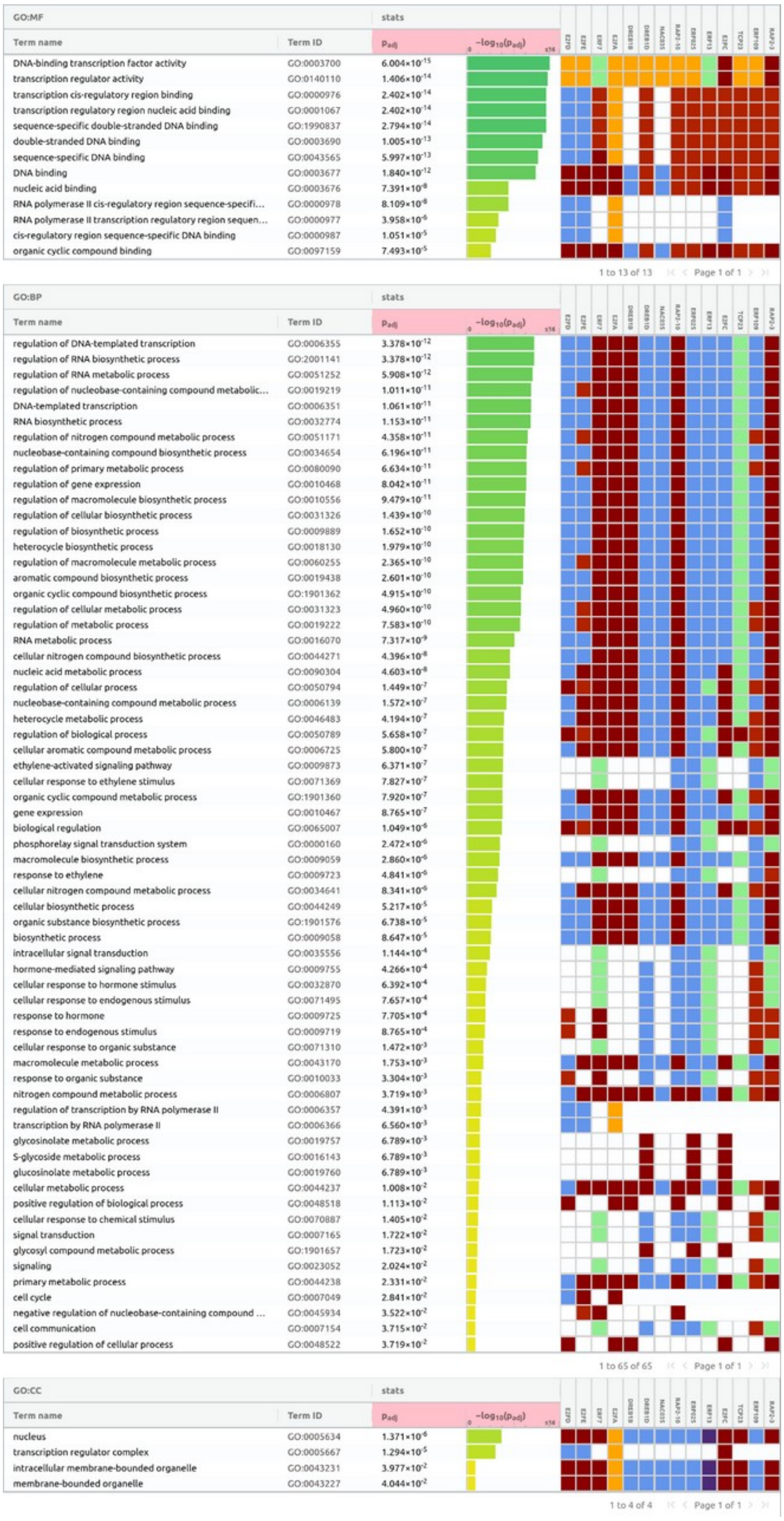

Supplementary Figure 13 - gProfiler GOSt analysis of the top 20 CNN model TFBS (LTR task).

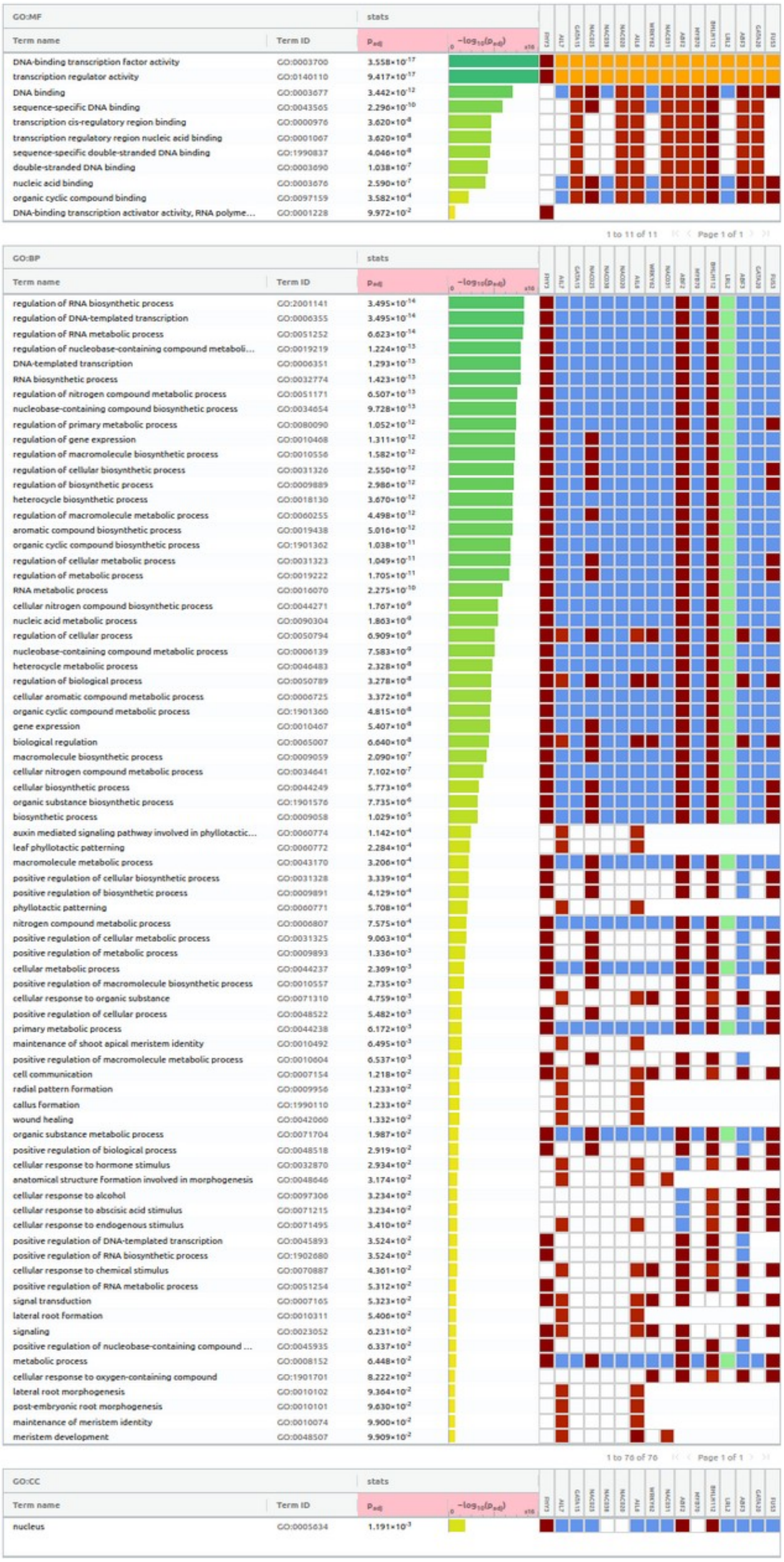

Supplementary Figure 14 - gProfiler GOSt analysis of the top 20 CNN model TFBS (superfamily task).

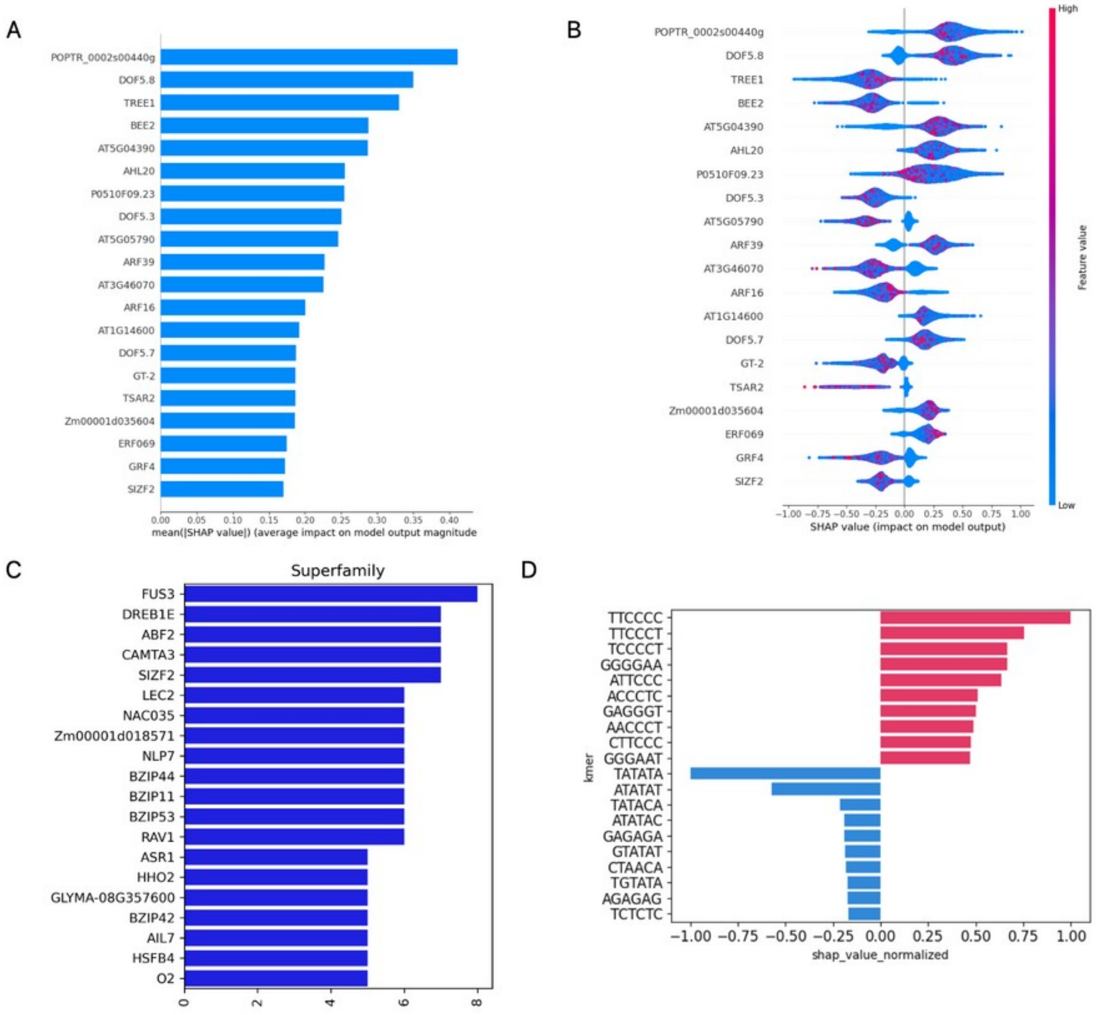

Supplementary Figure 15 - Main results of explainability analysis (superfamily classification). *A - Mean SHAP contribution of TFBS as input features to the GBC model for LTR classification. B - Beeswarm plot showing extent to which higher/lower values of input features influence model output. Negatively valued contributions are representative of the Copia superfamily, while positive contributions are representative of the Gypsy superfamily C - TomTom hits of first-layer CNN filters on JASPAR Core 2022 database D - Contribution of most significant k-mers used as input features for the DNABERT model. Values identical to those described for* ***B***

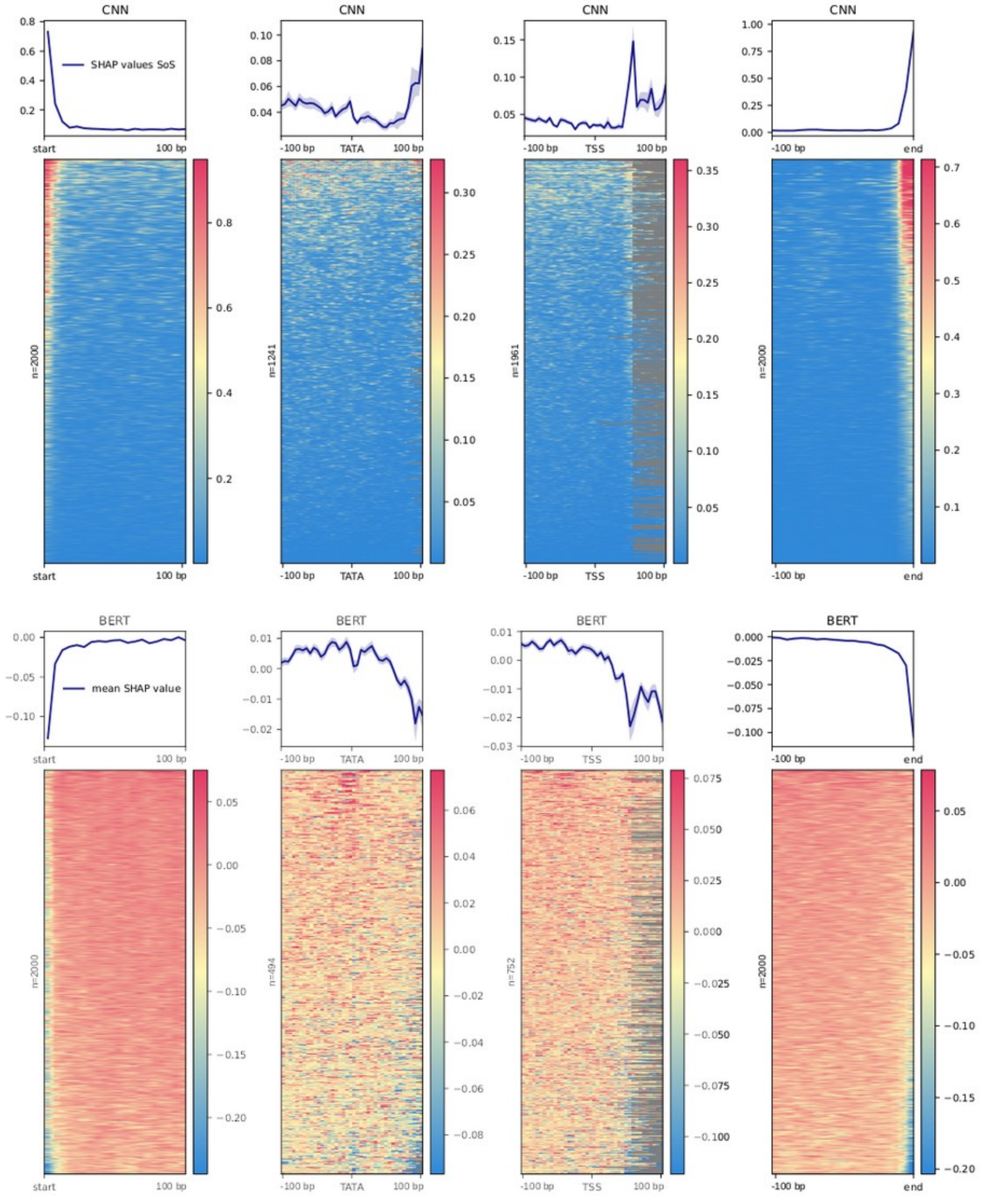

Supplementary Figure 16 - DeepExplainer analysis of trained superfamily detection models. *k-mer based SHAP values were calculated along individual LTR sequences. To visualize their alignment between different sequences, sequences were aligned (from left to right) by their first base (start), predicted TATA box (TATA), predicted transcription start site (TSS), or their last base (end). Averaged SHAP values are shown as a line graph above, individual sequence values are color-coded. A - CNN model. B - DNABERT model.*

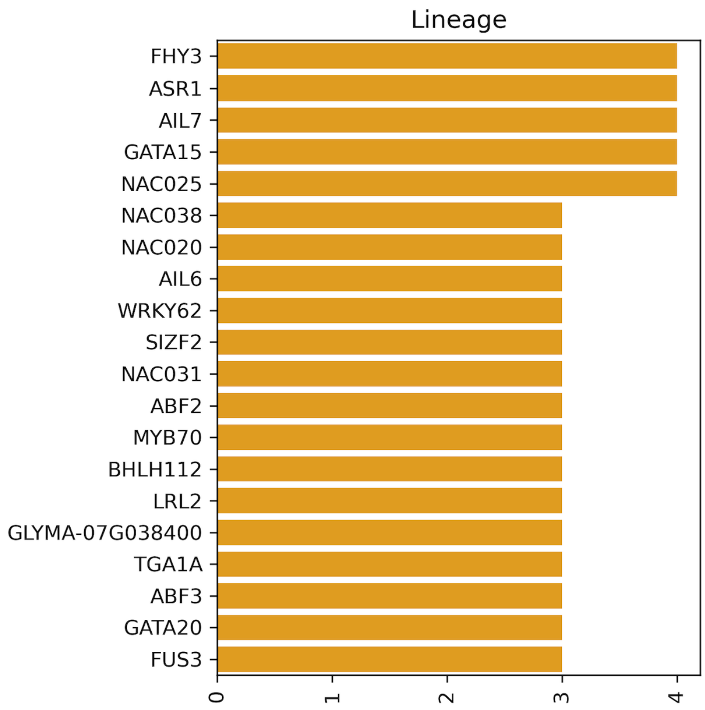

Supplementary Figure 17 - Results of explainability analysis (family classification). A - TomTom hits of first-layer CNN filters on JASPAR Core 2022 database.

Supplementary Tables

Model Type	Parameter	Tested values
Random Forest Classifier	N estimators	100, 300, 600
	Maximum depth	4, 6, 8, 10, 12
Multilayer Perceptron classifier	Hidden layer size	10, 50, 100, 200
	Learning rate	0.1, 0.05, 0.02, 0.01
	Alpha (Strength of L2)	0.0001, 0.001, 0.01
Gradient Boosting classifier	N estimators	50, 100, 200, 400
	Learning rate	0.1, 0.05, 0.02, 0.01
	Maximum depth	4, 6, 8
	Min samples at leaf node	20, 50, 100, 150

Supplementary Table 1 - Tested hyperparameters during grid search for each of the three models.

Classifier	Loss function
LTR classifier	Binary Crossentropy
Superfamily	Binary Crossentropy
Family	Categorical cross entropy

Supplementary Table 2 - Loss functions used for the different classification tasks.

	Comparison	Friedman p-value	Nemenyi p-value
LTR classification	CNN vs DNABERT	0.00003	0.53
	CNN vs GBC		0.008
	DNABERT vs GBC		0.001
Superfamily classification	CNN vs DNABERT	0.009	0.05
	CNN vs GBC		0.01
	DNABERT vs GBC		0.81
Family classification	CNN vs DNABERT	0.01	0.78
	CNN vs GBC		0.001
	DNABERT vs GBC		0.004

Supplementary Table 3 - Results of statistical testing on 10-fold cross-validation across all models. Friedman p-value - if $p < 0.05$ we reject the null hypothesis that the distribution of the scores is the same across all models. Nemenyi p-value - if $p < 0.05$ we reject the null hypothesis that there is no significant difference between the scores of the two models.

GBC

Task	N Estimators	Max Depth	Min Samples in Leaf Node
LTR	400	8	50
Superfamily	400	8	50
Family	400	8	50

CNN

Task	Filters	Filter Size	Dropout	LSTM Units	Optimizer
LTR	64	8	0.2	150	Adam
Superfamily	128	8	0.3	150	Adam
Family	128	16	0.2	150	Adam

BERT

Task	Filters	Filter Size	Dense Layer Size	Optimizer
LTR	128	3	64	Adam
Superfamily	128	3	64	Adam
Family	128	3	64	Adam

Supplementary Table 4 - Hyperparameter values used with trained models.
